# Alternative migratory tactics in brown trout (*Salmo trutta*) are underpinned by divergent regulation of metabolic but not neurological genes

**DOI:** 10.1002/ece3.7664

**Published:** 2021-06-02

**Authors:** Robert Wynne, Louise C. Archer, Stephen A. Hutton, Luke Harman, Patrick Gargan, Peter A. Moran, Eileen Dillane, Jamie Coughlan, Thomas F. Cross, Philip McGinnity, Thomas J. Colgan, Thomas E. Reed

**Affiliations:** ^1^ School of Biological, Earth and Environmental Sciences University College Cork Cork Ireland; ^2^ Environmental Research Institute University College Cork Cork Ireland; ^3^ Inland Fisheries Ireland Dublin Ireland; ^4^ Marine Institute Newport Ireland; ^5^ Present address: Department of Ecological Science – Animal Ecology Vrije Universiteit Amsterdam Amsterdam The Netherlands; ^6^ Present address: Institute of Organismic and Molecular Evolution Johannes Gutenberg University Mainz Mainz Germany

**Keywords:** alternative life histories, phenotypic plasticity, salmonids, sex bias, smoltification

## Abstract

The occurrence of alternative morphs within populations is common, but the underlying molecular mechanisms remain poorly understood. Many animals, for example, exhibit facultative migration, where two or more alternative migratory tactics (AMTs) coexist within populations. In certain salmonid species, some individuals remain in natal rivers all their lives, while others (in particular, females) migrate to sea for a period of marine growth. Here, we performed transcriptional profiling (“RNA‐seq”) of the brain and liver of male and female brown trout to understand the genes and processes that differentiate between migratory and residency morphs (AMT‐associated genes) and how they may differ in expression between the sexes. We found tissue‐specific differences with a greater number of genes expressed differentially in the liver (*n* = 867 genes) compared with the brain (*n* = 10) between the morphs. Genes with increased expression in resident livers were enriched for Gene Ontology terms associated with metabolic processes, highlighting key molecular–genetic pathways underlying the energetic requirements associated with divergent migratory tactics. In contrast, smolt‐biased genes were enriched for biological processes such as response to cytokines, suggestive of possible immune function differences between smolts and residents. Finally, we identified evidence of sex‐biased gene expression for AMT‐associated genes in the liver (*n* = 12) but not the brain. Collectively, our results provide insights into tissue‐specific gene expression underlying the production of alternative life histories within and between the sexes, and point toward a key role for metabolic processes in the liver in mediating divergent physiological trajectories of migrants versus residents.

## Graphical abstract

The occurrence of alternative morphs that differ in migratory tactics is common within populations, but the underlying molecular mechanisms remain poorly understood. Here, we used RNA‐seq to identify genes in the brain and liver that differ in expression between migrants and residents of the facultatively anadromous brown trout (*Salmo trutta*). We identified clear differences in the liver, particularly for genes enriched for metabolic process‐associated Gene Ontology terms, but not the brain, suggesting the importance of such genes in the regulation and expression of alternative migratory tactics.

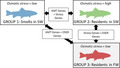

## 1 INTRODUCTION

Discrete phenotypic variation, where individuals from the same population develop into two or more morphs, is common in nature (Chevin & Lande, 2013; Roff, 1996). Developmental switches between alternative phenotypes, such as males versus females (Van Dooren & Leimar, 2003), insect castes (Schwander et al., 2010), trophic polymorphisms (Skulason & Smith, 1995), color polymorphisms (Roulin, 2004), horn polyphenisms (Moczek & Nijhout, 2002), or mating types (Brockmann & Tab orsky, 2008; Schunter et al., 2014), can reflect genetic or environmental determination, or some interaction between the two (Chevin & Lande, 2013; Roff, 1996; Tomkins & Hazel, 2007). Recent technical advances in genomics and bioinformatics (Alvarez et al., 2015; Todd et al., 2016) allow for novel insights into the molecular mechanisms and developmental plasticity underpinning the production of alternative phenotypes (Smith‐Gill, 1983; West‐Eberhard, 2003).

Alternative migratory tactics (AMTs), where both migratory and resident individuals coexist within a population (also known as partial, or facultative, migration), represent a particularly interesting type of discrete phenotypic variation that occurs in all major vertebrate groups and many invertebrates (Chapman et al., 2011). The adoption of AMTs is triggered by environmental cues in interaction with genetically inherited thresholds, with various selective mechanisms involved in the maintenance of AMTs and associated phenotypic variation across time and space (Buoro et al., 2012; Pulido, 2011; Tomkins & Hazel, 2007). Anthropogenic influences can also drive rapid shifts in AMT frequencies via microevolution or phenotypic plasticity (McCleave & Edeline, 2009; Phillis et al., 2016; Pulido, 2007; Thériault et al., 2008); hence, understanding the proximate and ultimate drivers of migration versus residency is a topic of increasing conservation and management relevance (Railsback et al., 2014).

Diadromous migrations between fresh and saltwater habitats occur in many fishes (McDowall, 1997), which, on top of the rigors of migration itself, involve the added challenge of coping with transitions between different osmotic environments. For example, salmonid fishes (salmon, trout, charr, and relatives) often undertake anadromous migrations between freshwater spawning and marine feeding habitats. Species, populations, and individuals vary markedly in rates of anadromy, as well as in the spatial extent and duration of marine migrations (Klemetsen et al., 2003; Quinn & Myers, 2004). In populations where both anadromous and resident individuals occur, they freely interbreed and offspring inherit a propensity for one or other tactic as a polygenic threshold trait (Dodson et al., 2013; Ferguson et al., 2019; Kendall et al., 2015; Sloat et al., 2014). The benefits of anadromy, which include enhanced growth in the marine environment and avoidance of seasonally harsh freshwater conditions, are finely balanced against the costs, which include increased energetic expenditure, potentially greater mortality risks, and less scope for early maturation (Curry et al., 2010; Dodson et al., 2013; Ferguson, 2017; Ferguson et al., 2019; Hendry & Stearns, 2004; Kendall et al., 2015; Nevoux et al., 2019; Pavlov & Savvaitova, 2008; Sloat & Reeves, 2014). The life history trade‐offs involved also vary between the sexes, with females typically gaining more in reproductive success terms from the larger potential body sizes afforded by marine feeding. Females are thus more likely to adopt an anadromy tactic, and males tend to retain a residency tactic, with males also exhibiting a broader range of ages and sizes at first reproduction (Jonsson & Jonsson, 1993).

Many genes are likely involved in the initial decision to adopt a migratory instead of a residency tactic (Hecht et al., 2013, 2015), potentially including some major effect loci (reviewed by Ferguson et al., 2019). Studies on rainbow/steelhead trout (*Oncorhynchus mykiss*), for example, have identified a migration‐associated region (MAR) on chromosome *Omy5* that might harbor a “master control switch” for AMTs (Arostegui et al., 2019; Kelson et al., 2019; Leitwein et al., 2016; Nichols et al., 2008; Pearse & Campbell, 2018). This MAR involves a 55‐Mb double inversion that acts as a “supergene” influencing sex‐specific migratory tendencies via sex‐dependent dominance (Pearse et al., 2019). Once an individual has “committed” to a particular AMT—a decision that might occur months or even years in advance of actual migration, depending on the species—a series of molecular changes are set in motion that channel residents and (future) migrants along divergent developmental trajectories. Migrants and residents can only be differentiated externally at the smoltification stage, when migrants undergo morphological and physiological transformations in preparation for transition to the marine environment. However, internal differences in patterns of energy acquisition and allocation to competing functions, such as metabolism, growth, and lipid storage, are apparent much earlier in the life history (reviewed in Dodson et al., 2013; Ferguson et al., 2019).

Complex gene regulatory mechanisms, perhaps involving one or more master regulators (Aubin‐Horth et al., 2009) and a series of epigenetic modifications (Baerwald et al., 2016), likely control this phenotypic divergence. Theory on the evolution of developmental plasticity indeed suggests that morph‐specific gene expression can decouple developmental pathways and reduce pleiotropic correlations among traits expressed in each morph (Snell‐Rood et al., 2010). For example, in *O. mykiss,* differential gene expression between migrant and resident offspring is evident in the brain a full year before smoltification (McKinney et al., 2015). As smoltification approaches, migrants undergo phenotypic “remodeling,” including changes in body shape and color, behavioral changes, and hormonal plus enzyme alterations (McCormick, 2012) that are underpinned by transcriptomic differences (Aykanat et al., 2011; Houde et al., 2019; Sutherland et al., 2014). Complex genetic networks are involved that might include clusters of genes on particular chromosomes, for example, *Omy12* in *O. mykiss* (Hecht et al., 2012, 2013). Environmental‐dependent methylation changes might also play a regulatory role in seawater acclimation of smolts (Morán et al., 2013). Residents, in contrast, increasingly allocate more energy and resources to maturation processes, with males in particular often maturing earlier than (resident or anadromous) females (Archer et al., 2019; Archer, Hutton, Harman, McCormick, et al., 2020; Archer, Hutton, Harman, Poole, et al., 2020; Jonsson & Jonsson, 1993; McMillan et al., 2012).

Here, we use RNA sequencing (“RNA‐seq”) to explore differential gene expression between migrant and resident brown trout (*Salmo trutta*), a species exhibiting facultative anadromy and flexible freshwater migration strategies (Ferguson, 2017; Ferguson et al., 2019; Nevoux et al., 2019). Previous studies of AMTs in salmonids have focused on the brain as a key organ that might regulate divergent phenotypic trajectories by integrating internal and external signals, and found differential expression in genes associated with metabolism, morphology, olfactory imprinting, osmoregulation, and sexual maturation (Hale et al., 2016, 2018; McKinney et al., 2015). In brown trout, genomic studies have found loci associated with variation in the distance of migration (Lemopoulos et al., 2018, 2019), while previous transcriptomic work using cDNA microarrays with a restricted number of genes identified gene expression differences in the liver associated with migratory versus sedentary lifestyles (Giger et al., 2006, 2008). We therefore focused on the liver and brain as candidate organs mediating AMTs and smoltification processes in brown trout. Our first objective was to identify genes that are differentially expressed between individuals classified as immature smolts (anadromous migratory tactic) or mature nonsmolts (residents, i.e., freshwater maturation tactic), here defined as putative “AMT‐associated genes.” To distinguish these from genes that are differentially expressed as a result of the general stress of a transition from fresh to salt water, we compared putative smolts against both freshwater and saltwater‐exposed mature individuals (i.e., residents). Second, we examined whether putative AMT‐associated genes demonstrated conserved or tissue‐specific expression profiles. Lastly, we asked whether genes differentially expressed between smolts and residents were also differentially expressed between males and females, given their divergent phenotypic trajectories with respect to migration.

## 2 MATERIALS AND METHODS

### 2.1 Laboratory rearing of fish

Fish in our study were reared from the egg stage in a controlled laboratory environment as part of a broader study on genetic and environmental drivers of facultative anadromy (Archer et al., 2019; Archer, Hutton, Harman, McCormick, et al., 2020; Archer, Hutton, Harman, Poole, et al., 2020). Here, we sampled fish from a single population background and environment of rearing (i.e., single rearing tank; see Archer et al., 2019; Archer, Hutton, Harman, McCormick, et al., 2020; Archer, Hutton, Harman, Poole, et al., 2020, for full details on fish husbandry) to avoid confounding effects in the gene expression data. The sampled fish represent the offspring of four wild‐caught females and five wild‐caught males, with each female mated to two males; thus, we had eight full‐sibling families nested within four maternal half‐sibling families.

The wild‐born parents were caught in November 2015 in Tawnyard Lough, a small upland lake in the western reaches of the Erriff catchment in the west of Ireland (53°37′0.00″N: 09°40′17.10″W). The lake is fed primarily by the Glendavoch River, within which there is extensive suitable spawning habitat for *Salmo trutta*, along with a series of smaller spawning tributaries. Tawnyard Lough produces a large run of anadromous smolts annually (Gargan et al., 2016), but some juveniles remain within the lake and never go to sea, instead maturing in freshwater. It was not possible to distinguish clearly whether the wild‐born parents in our study had been to sea or not, but the Tawnyard population, in general, shows high rates of anadromy (Archer et al., 2019; Archer, Hutton, Harman, McCormick, et al., 2020; Archer, Hutton, Harman, Poole, et al., 2020; Gargan et al., 2016). Thus, we expected a genetic predisposition toward high rates of smolting in our tank‐reared fish, but also for some individuals to adopt a freshwater maturation (residency) tactic.

### 2.2 Determination of smolt status

Over 22 months of tank rearing, 74 fish were routinely assessed for morphological indicators of smoltification, following the criteria of Tanguy et al. (1994). Smolts undergo a change in coloration from dark to silvered flanks, lose color in the fins, and lose their parr marks (the dark traverse bands on the side of young salmonids). They also have pronounced lateral lines and a more fusiform body shape than parr (the juvenile freshwater stage prior to smoltification of migrants). Sixteen putative smolts were thus identified in early summer 2018 (at age 2+). Such morphological indicators are not always a reliable guide to saltwater tolerance capabilities in this highly phenotypically variable species (Klemetsen, 2013). We therefore subjected our putative smolts to a saltwater tolerance test to assess their hypo‐osmoregulation capacities. Fifteen putative residents (fish showing no outward signs of smolting) were also subjected to the same saltwater tolerance test, to confirm their nonsmolt status.

The trials involved transferring fish from their freshwater tank into another tank at a salinity of 30 parts per thousand (ppt) for 24 hr, a period sufficient to induce hypo‐osmoregulation in euryhaline species (Schultz & McCormick, 2012), and then measuring their plasma chloride levels. Low plasma chloride concentrations indicate physiological tolerance to salt water (full details provided in Archer et al., 2019; Archer, Hutton, Harman, McCormick, et al., 2020; Archer, Hutton, Harman, Poole, et al., 2020). A salinity of 30 ppt was used rather than full oceanic salinity (35–40 ppt) to minimize unnecessary stress on the fish, and also because wild brown trout often spend large amounts of time in estuaries during seaward migration (Ferguson et al., 2019; Nevoux et al., 2019). Immediately after 24‐hr saltwater exposure, the fish were euthanized with an overdose of *Tricaine mesylate* (MS‐222) and a blood sample was collected from the caudal vein using a 21‐G needle and a 2.6‐ml heparinized syringe. Blood samples were transferred to 2‐ml Eppendorf tubes and centrifuged at 8,000 x *g* for 3 min to separate the plasma from the erythrocytes and other cellular components. The plasma supernatant was then siphoned off via pipette into a new 1‐ml Eppendorf tube and stored at −80°C before being measured for plasma chloride concentration (mmol/L) by coulometric titration using a Jenway PCLM3 chloride meter (FishVet Group, Oranmore, Ireland).

**FIGURE 1:**
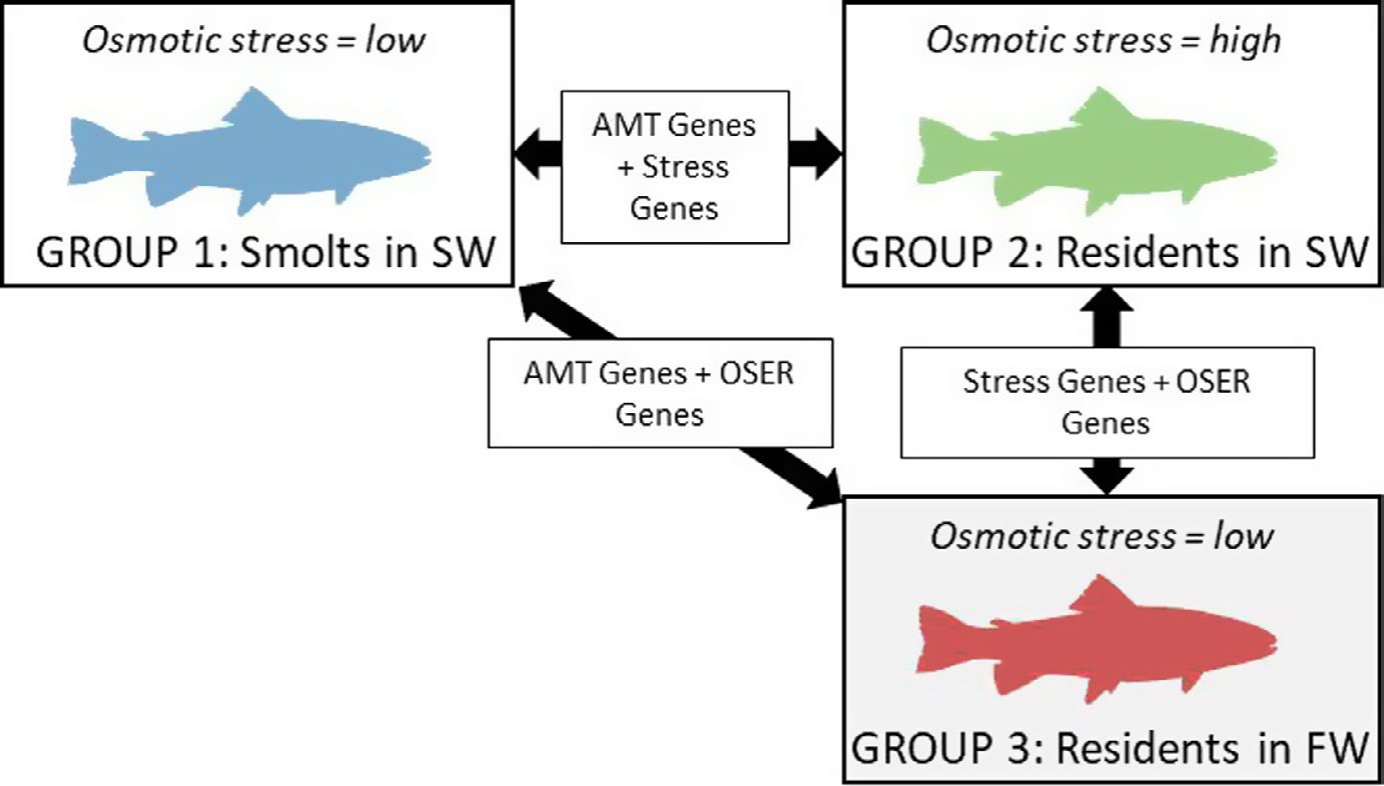
Identification of putative AMT genes in brown trout. Panel schematic representation of experimental design and associated logic. Livers and brains of trout deemed to have adopted an anadromous tactic (smolts) were RNA‐sampled immediately following a 24‐hr saltwater (SW) challenge (group 1 = smolt‐SW). Livers and brains of a second group of trout deemed to have adopted a resident tactic were sampled following the same SW challenge (group 2 = resident‐SW), while those of a third group were sampled at the same time in freshwater (FW) without having undergone a saltwater challenge (group 3 = resident‐FW). Note that smolts could not be tested in FW prior to being subjected to a SW challenge (which is required to define a fish as a smolt or not) because liver and brain sampling is terminal for the fish. Because smolts are by definition physiologically tolerant of SW, group 1 experienced mild osmotic stress. Residents are physiologically intolerant of SW but well suited to life in FW; hence, group 2 experienced high osmotic stress, while group 3 experience low osmotic stress. Black arrows represent genes that are differentially expressed between each pairwise comparison. AMT genes refer to those that are differentially expressed between the alternative life history tactics and potentially encompass genes involved in morphology, behavior, physiology (including saltwater tolerance), and reproduction. Stress genes here refer to those for which differential expression between osmotic environments reflects the general stress of transitioning from FW to SW. Here, OSER genes (osmotic environmental response genes) refer to genes that would be differentially expressed in response to different environmental salinity

Given their expected poorer hypo‐osmoregulatory abilities, residents were expected to experience higher stress levels than smolts as a result of this acute exposure to salt water. Thus, comparing gene expression differences between smolts and residents after saltwater testing might confound life history (AMT) differences with stress differences (Figure 1). To explore this, we also sampled (see below) an additional group of residents (*n* = 9) who were not subjected to the saltwater challenge but remained within their original freshwater tank and hence experienced similar, or slightly lower, stress levels than the smolts (who might have experienced mild osmotic stress during the saltwater trial).

Fish from all three groups (putative smolts exposed to salt water, putative residents exposed to salt water, and putative residents not exposed to salt water) were terminally sampled in early summer 2018 and dissected to determine maturation status and extract liver and brain tissue for transcriptional profiling. Before tissue extraction, every fish was measured for fork length (to the nearest mm) and weight (to the nearest g) and a small section of tail fin tissue was removed using a sterilized scalpel and stored in 100% ethanol for subsequent DNA analysis (to determine genotypic sex, see below). Each dissection session took place at the same time each morning in an attempt to minimize among‐individual variation in gene expression related to circadian rhythms. Visual gonad inspections were performed first, and then, whole brains and livers were removed and transferred into individual sterile 2‐ml Eppendorf tubes containing 1ml of RNAlater solution. Samples were incubated at 4°C for 24 hr to allow RNAlater to permeate through tissues, and then transferred to −80°C storage.

Females were classified as sexually mature if the body cavity was filled with identifiable eggs, whereas males were classed as sexually mature if they had enlarged, white testes, or were running milt. A fish was classified as immature if gonads were undeveloped (see Archer et al., 2019 for full details). This maturation status information, coupled with the morphological and physiological evidence for smolting, then allowed us to define three final groups of individuals for whom smolt status (morphological signs of smolting present, low plasma chloride, and reproductively immature) or resident status (morphological signs of smolting absent, high plasma chloride if salt water tested, and reproductively mature) could be confidently assigned. From this, we concluded that seven of the saltwater‐tested individuals could be classified as smolts. This resulted in a final sample set consisting of seven saltwater‐tested smolts (“smolt‐SW”; total *n* = 7:3 males and 4 females), nine saltwater‐tested residents (“resident‐SW”; *n* = 9:5 males and 4 females), and nine freshwater (non‐saltwater‐tested) residents (“resident‐FW”; *n* = 9:5 males and 4 females). Fish in our smolt‐SW group had lower average plasma chloride concentrations (mean = 129.6 mmol/L, standard deviation, *SD* = 12.6) compared with fish in our resident‐SW group (mean = 159.2 mmol/L, *SD* = 13.9; unpaired *t* test: *t* = 4.4, *p* < .001, *df* = 13.49), confirming the former had adopted a migratory tactic.

### 2.3 RNA extractions

Total RNA was extracted from each individual brain and liver sample using TRI Reagent (Sigma‐Aldrich) and subsequently purified with GenElute™ Mammalian Total RNA MiniPrep Kits (Sigma‐Aldrich), following manufacturer's instructions. We DNase‐treated each sample using the Turbo DNA‐free Kit (Qiagen, UK) to remove residual DNA. We initially assessed the quality and quantity of purified RNA using the NanoDrop 2000 (Thermo Fisher, USA) and the Qubit RNA Broad Range Assay Kit with a Qubit 4 Fluorometer (Thermo Fisher, USA), respectively. Finally, we calculated a relative integrity number (RIN) per sample using an Agilent TapeStation 4200 (Agilent, USA) to ensure purified RNA was not degraded and we only sent samples with a RIN of seven or higher (Sigurgeirsson et al., 2014) for subsequent library preparation and sequencing.

### 2.4 Library preparation and sequencing

We shipped samples (*n* = 49; 24 livers and 25 brains) to Novogene Europe (Cambridge, UK) for mRNA‐enriched library preparation and sequencing. In brief, DNase‐treated RNA quality was secondarily assessed by TapeStation at Novogene to ensure quality was high and no degradation occurred during transport. For each sample, an individual mRNA‐enriched library was generated using the NEBNext Ultra II RNA library preparation kit (NEB, UK). Individual libraries were barcoded with specific identifiers and multiplexed, and sequencing (150bp paired‐end (PE)) was performed on an Illumina NovaSeq 6000 generating a total of ~1.29 billion PE reads. For liver samples, the total number of PE reads was ~590 million (mean: ~24.6 million, min: ~20.3 million, max: ~35.4 million), while for brain samples, ~699 million reads were generated (mean: ~27.9, min: ~20.4 million, max: ~42.4 million). Sample information, including the total number of paired‐end reads generated per sample, is provided in Table S1.

### 2.5 Sequence data quality assessment

To assess the quality of our raw reads, we used FastQC (version 0.11.3; Andrews, 2010) to identify the presence of sequences with low base quality and/or adapter contamination. Overall, FastQC indicated high sequence quality. Following this, we estimated transcript abundance for each sample through quasi‐aligning reads with Salmon (version 1.2.0; Patro et al., 2017) against predicted transcripts (cDNA sequences) from the publicly available *Salmo trutta* reference genome assembly (fSalTru1.1, INSC Assembly GCA_9010011651.1) obtained from Ensembl. Using these estimates, we generated summarized gene‐level counts per individual sample using tximport (version 3.10; Soneson et al., 2015), which were then imported into DESeq2 (version 3.10; Love et al., 2014) for differential expression analysis. For the purpose of reanalysis, gene‐level counts are provided in Table S2. For differential expression and Gene Ontology enrichment analyses, we used modified scripts initially developed by Colgan et al. (2019).

### 2.6 Differential expression analyses

To first get a broad sense of global differences in transcriptional profiles across our three groups, we ran a principal component analysis (PCA) on variance stabilizing transformed gene‐level counts using DESeq2. Given expected differences in transcriptional profiles between tissues, we performed independent PCAs for liver and brain samples using the plotPCA function in DESeq2.

For each tissue, we next performed gene‐level tests for differential expression between comparisons of interest using DESeq2. We first defined a global model with main effects of “phenotype”—a two‐level factor indicating whether the individual fish was a smolt or resident—and “testing environment”—a two‐level factor indicating whether the fish was exposed to the 24‐hr salt water test just prior to terminal sampling (salt), or not (fresh), as explanatory variables. Note that phenotype and testing environment are not perfectly crossed, as all smolts were saltwater‐tested (which was necessary to confirm their smolt status in physiological terms), whereas the residents fell under either salt or fresh for testing environment (Figure 1). This global model was then compared against two reduced models, one that contained only a main effect of environment and one that contained only a main effect of phenotype. This enabled us to generate *p* values, on a gene‐by‐gene basis, for the effects of phenotype and environment. We then defined differentially expressed genes as those where the absolute log2 fold change was greater than one, and the Benjamini–Hochberg‐adjusted *p* values were less than .05. This therefore yielded two lists of genes: those differentially expressed between smolts and residents, and those differentially expressed between saltwater versus freshwater testing environment.

To isolate the effect of phenotype per se, we identified the unique subset of genes that were differentially expressed between smolts and residents only and not also between osmotic testing environments. Hereafter, we refer to these genes as “AMT‐associated genes.” Similarly, genes that were differentially expressed between saltwater versus freshwater testing environments, but not also between smolts and residents, were classified as “osmotic environment response genes.” Genes that were differentially expressed between both comparisons were labeled as “stress genes.” For each set of genes, we also used heatmaps and scatter plots (using the ggplot2 package, version 3.3.2 in R) to explore and illustrate the patterns of (z‐score‐normalized) gene expression with respect to our three groups, that is, smolt‐SW, resident‐SW, and resident‐FW, for livers and brains separately.

Finally, we tested for each tissue whether any of our AMT‐associated genes or osmotic environment response genes were also differentially expressed between males and females. To achieve this, a DESeq2 model with a main effect of sex was compared against a null model, with no effects. We defined significantly differentially expressed genes as before (i.e., absolute logFC >1 between males and females and BH‐adjusted *p* < .05). We then identified which of these sex‐biased genes had also been classified as AMT‐associated genes or osmotic environment response genes in the above analyses.

### 2.7 Gene Ontology term enrichment analyses

For each gene in the *S*. *trutta* reference genome, we used the functional annotations assigned to the corresponding orthologs in the *Danio rerio* (zebrafish) genome, obtained from Ensembl BioMart (Kinsella et al., 2011), as very little functional information exists for brown trout genes. For both the smolt‐biased and resident‐biased AMT‐associated genes, we tested for enrichment of Gene Ontology (GO) terms using Fisher's exact tests in topGO (version 3.10; Alexa et al., 2006), applying the “weight01” algorithm and a node size of 50. We corrected for multiple testing using the Benjamini–Hochberg method to generate adjusted *P* values. As a means of understanding more general processes enriched with differentially expressed genes, we also subset higher level GO terms of significantly enriched GO terms (e.g., level‐two GO terms that are “child” terms of each of the three main GO subcategories of “Biological Process,” “Molecular Function,” and “Cellular Component”).

## 3 RESULTS

### 3.1 Distinct AMT‐associated transcriptional profile in liver but not brain

**FIGURE 2:**
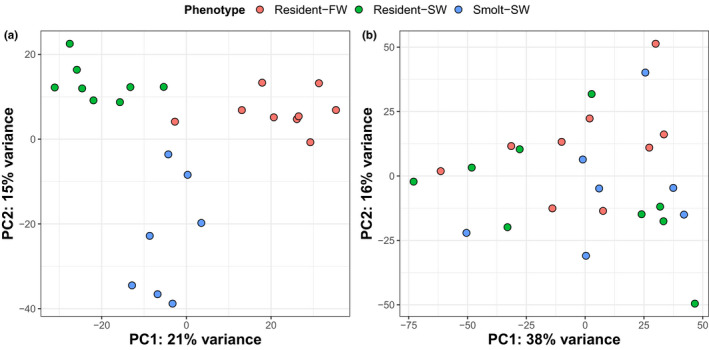
Distinct transcriptional profiles between resident and smolt livers but not brains. Scatterplots displaying the results of a principal component analysis (PCA) for normalized gene expression values for (a) livers and (b) brains collected from three groups differing in phenotype and/or environment. The proportion of variance within gene expression explained by principal component 1 (PC1) and 2 (PC2) is displayed on the *x*‐axis and *y*‐axis, respectively. Here, each group is a categorical variable corresponding to the combination of the individual phenotype (life history tactic) and osmotic environment the individual experienced for 24 hr just prior to terminal sampling (salt water or fresh water)

For the livers, PCA demonstrated a clear separation of samples based on gene expression with respect to our three groups, that is, combinations of phenotype and testing environment (Figure 2a). The first principal component (PC1) accounted for 21% of the total variance and primarily separated resident‐SW individuals, who likely experienced high osmotic stress, from resident‐FW, who presumably experienced lower osmotic stress, with smolt‐SW individuals intermediate. In contrast, PC2, which accounted for 15% of the total variance, primarily separated smolt‐SW individuals from residents (Figure 2a). The combination of PC1 and PC2 separates smolts from residents, with smolts having high values for both PC1 and PC2, but residents having lower PC2 and a range of PC1 values. In contrast, the PCA on the brain samples did not produce clear separation among the three groups based on the first two principal components (Figure 2b), nor PC3‐6 (result not shown).

### 3.2 Liver AMT‐associated genes are enriched for metabolic process‐related GO terms

We identified 1,670 genes that were significantly differentially expressed (LRT, BH‐adjusted *p* < .05) between the livers of smolts and residents. A total of 3,426 genes were differentially expressed between fish sampled in saltwater and freshwater testing environments (global model vs. reduced model with phenotype effect only). We found 867 genes that were uniquely differentially expressed between the phenotypes but not osmotic testing environments, which we designate as potential AMT‐associated genes. The output of both models, as well as the different subcategories of genes, is provided in Table S3.

**FIGURE 3:**
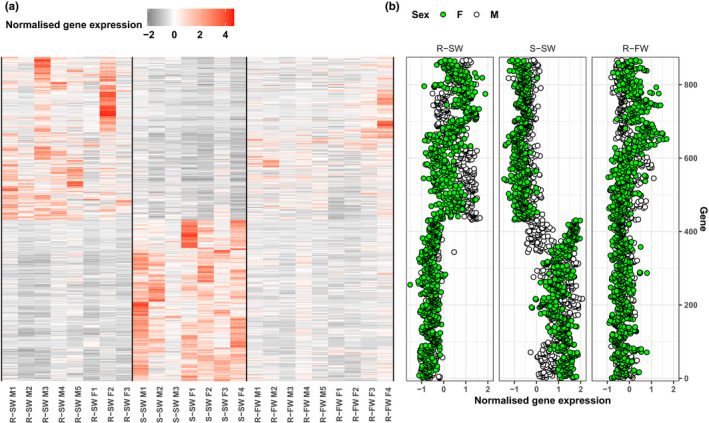
Putative AMT genes expressed in the brown trout liver. (a) Heatmap displaying the normalized gene‐level expression estimates for 867 genes that were uniquely differentially expressed between the livers of resident and migrant brown trout. Each row represents a single gene (*y*‐axis), while for each sample (*x*‐axis), the sample name provides information on phenotype (R = “resident,” S = “smolt”), environment of sampling (SW = “salt water,” FW = “freshwater”), and sex (*M* = “male,” *F* = “female). (b) Scatterplots showing the mean normalized gene expression for each sex within each of three groups (R‐SW = “resident salt water,” S‐SW = “smolt salt water,” R‐FW = “resident fresh water”). Each point represents an individual gene and is colored by sex (green = “female,” white = “male”)

**FIGURE 4:**
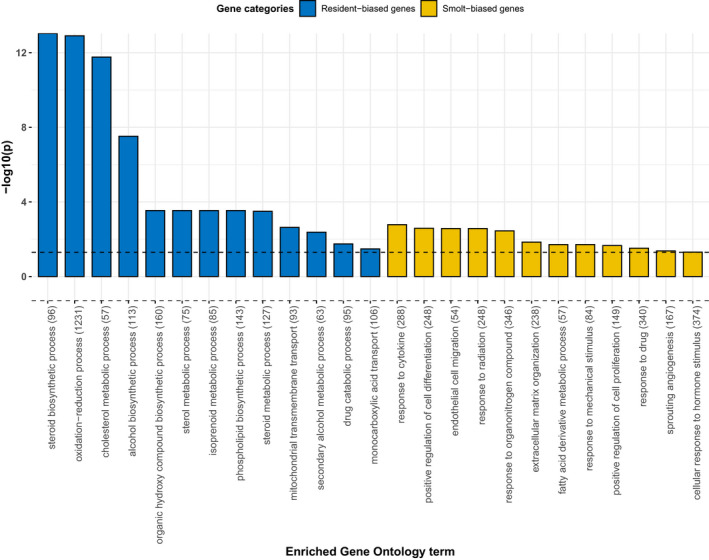
Gene Ontology enrichment analysis of putative AMT genes in brown trout liver. Enriched Gene Ontology associated with biological process‐related terms for genes differentially expressed between alternative life histories. Each bar represents the −log_10_ of adjusted *p* value (Fisher's exact test) for each GO term with description of GO term, as well as the total number of annotated genes per term provided. The black vertical dashed line represents a −log_10_(*p*) value equivalent to an adjusted *p* value = .05 threshold of significance

Of the 867 putative AMT‐associated genes (Figure 3), we identified 430 genes with increased expression in residents relative to smolts, while 437 genes showed increased expression in smolts relative to residents (Table S3). The resident‐biased genes were enriched (BH‐adjusted *p* < .05) for 21 GO terms, including 13 terms related to “biological process” (Figure 4) with the majority categorized under “metabolic processes” (Table S4). The three most significant terms were steroid biosynthesis process (GO:0006694), oxidation‐reduction process (GO:0055114), and cholesterol metabolic process (GO:0008610) (Table S4). In contrast, the 437 genes with smolt‐biased expression were significantly enriched for 14 GO terms. The majority of these enriched terms (*n* = 12) were “biological process”‐related GO terms with the three most significant GO terms being response to cytokine (GO:0034097), positive regulation of cell differentiation (GO:0045597), and endothelial cell migration (GO:0043542) (Figure 4). These terms form part of GO term hierarchies with higher level terms, such as cellular processes, response to stimulus, and metabolic processes (Table S4).

### 3.3 Fewer transcriptional changes in brain underlying AMTs

We identified 53 genes that were significantly differentially expressed between the brains of smolts and residents (Table S3C). We found 716 genes that were differentially expressed between saltwater and freshwater testing environments (Table S3D), and 43 genes that overlapped between both comparisons. Thus, ten genes were uniquely differentially expressed between the phenotypes but not testing environments (i.e., brain AMT‐associated genes).

Seven of these genes exhibited elevated expression in smolts compared with residents: Sox18 (ENSSTUG00000006705), protein phosphatase PTC7 (ENSSTUG00000008660), E3 ubiquitin‐protein ligase RNF38‐like (ENSSTUG00000023791), N‐acetyltransferase 8‐like gene (ENSSTUG00000014735), BUB3 mitotic checkpoint protein (ENSSTUG00000036930), a zinc finger protein 239‐like (ENSSTUG00000002250), and an uncharacterized gene (ENSSTUG00000047265). There is an annotated ortholog for this latter gene in *Salmo salar* where it has been described as an ankyrin‐3‐like (ENSSSAG00000039215). The three genes with increased expression in the brains of residents compared with smolts were a zinc finger and BTB domain‐containing protein (ENSSTUG00000030799), sperm tail PG‐rich repeat containing protein (ENSSTUG00000039823), and plexin‐A1 (ENSSTUG00000046884). These genes were not significantly enriched (BH‐adjusted *p* > .05) for any specific GO term. Furthermore, no overlap was identified between brain and liver AMT‐associated genes.

### 3.4 Sex‐biased gene expression in certain AMT‐associated genes

**FIGURE 5:**
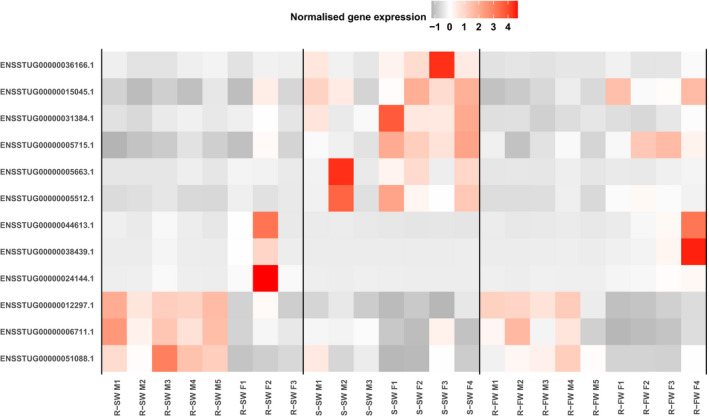
Sex‐biased expression of putative AMT genes. Heatmap displaying the normalized gene‐level expression estimates for 12 putative AMT genes that also demonstrated sex‐biased gene expression within the liver. For each gene, the Ensembl gene ID is provided (*y*‐axis), while for each sample (*x*‐axis), the sample name provides information on phenotype (R = “resident,” S = “smolt”), environment of sampling (SW = “salt water,” FW = “fresh water”), and sex (*M* = “male,” *F* = “female”)

We identified evidence of sex‐biased gene expression in the liver with 103 genes significantly differentially expressed between males and females (Table S5). Of these genes, 12 were also differentially expressed between smolts and residents. We found no significant enrichment (BH‐adjusted *p* > .05) for GO terms for these 12 genes. The analyzed transcriptome as a whole comprised 44,366 transcribed genes, of which 0.23% (103/44,366) exhibited sex‐biased expression, whereas just over 1% of our AMT‐associated liver genes (12/867) exhibited sex‐biased expression. Thus, there was a statistically significant excess of sex‐biased AMT‐associated genes in comparison with the overall proportion of sex‐biased genes genome‐wide (chi‐squared = 45.7, *df* = 1, *p* < .001). Among these 12 sex‐biased AMT‐associated liver genes, six exhibited elevated expression in residents relative to smolts and six demonstrated smolt‐biased expression. The majority of these genes (*n* = 9) were also elevated in females relative to males, with the sex differences being typically larger in smolts than residents (Figure 5). The remaining three sex‐biased AMT‐associated liver genes had higher expression in male residents than in female residents.

We identified eight genes in the brain that exhibited sex‐biased gene expression. However, we found no overlap between these genes and the ten AMT‐associated genes that differed between the brains of smolts and residents.

## 4 DISCUSSION

Here, we compared whole‐genome transcriptomic profiles of sea‐migratory versus sea‐resident brown trout during the presumed migration period, to investigate molecular processes underpinning the generation of well‐integrated, ecologically important, alternative phenotypes. Our study revealed three key findings: (a) extensive transcriptional differences between migratory and resident brown trout for genes underlying metabolic, cellular, and immune processes (particularly genes enriched for GO terms associated with metabolic processes, which had the lowest adjusted *p* values; Figure 4); (b) tissue differences in expression profiles between migrants and residents with distinct life stage‐specific patterns evident in the liver but not brain; and (c) sex‐biased expression differences in genes associated with AMTs. The genes we identify may be downstream targets of master regulatory genes proposed to trigger AMTs and allow for the transition between aquatic environments; alternatively, they may represent nontarget effects of physiological interactions unrelated to migration.

Animal migration can take place over long distances and last prolonged periods requiring dynamic changes in behavioral, physiological, morphological, and biochemical aspects of an organism's phenotype. In metabolic terms, migration utilizes substantial energetic resources (Wikelski et al., 2003), which likely require tight regulation during such extended periods of metabolic stress. A key organ involved in such regulation is the liver. Here, we identified distinct transcriptional profiles between migrants and residents with genes differentially expressed between these alternative phenotypes enriched for Gene Ontology terms involved in a range of key metabolic processes (Figure 4), including steroid biosynthesis, cholesterol biosynthesis, phospholipid, and fatty acid metabolism, with a general reduction in expression of such genes within migrants compared with residents. These findings are in line with previous molecular and biochemical research performed on other salmonids that identified changes in glycogen stores due to the energetic demands of migration, as well as changes in gene expression underlying lipid remodeling during transition from a freshwater to a saltwater environment (Gillard et al., 2018). On top of changes in genes associated with lipid metabolism, we also identified additional metabolic processes that differ between migrants and residents. Such changes may also be related to differences in reproductive maturity status, whereby energetic demands vary due to residents being fully mature reproductively, while for migrants (who were the same age but immature), reduced expression of metabolic genes may be associated with suppressed reproductive development.

The above findings tally with previous work on brown trout, and on other salmonids exhibiting AMTs, that has established links between various aspects of an individual's physiological condition and its migratory status (reviewed by Dodson et al., 2013; Ferguson et al., 2017, 2019; Kendall et al., 2015; Sloat et al., 2014). For example, energy limitation owing to poor food availability is associated with higher rates, or earlier ages, of migration (Archer et al., 2019; Archer, Hutton, Harman, McCormick, et al., 2020; Archer, Hutton, Harman, Poole, et al., 2020; Jones et al., 2015; Morán et al., 2013; Olsson et al., 2006; O'Neal & Stanford, 2011; Shry et al., 2019; Wysujack et al., 2009), and the energy value of food may also be important (Kendall et al., 2015). Metabolic rate, and how it links to growth efficiency and energy allocation to competing functions (e.g., lipid storage; Boel et al., 2014; Jonsson & Jonsson, 2005), is further believed to underpin these associations (Archer, Hutton, Harman, McCormick, et al., 2020; Archer, Hutton, Harman, Poole, et al., 2020; Forseth et al., 1999; McCarthy, 2000; Norin & Malte, 2011; Seppänen et al., 2010; Sloat & Reeves, 2014). Our study found that the most differentially expressed gene between smolts and residents was *serine/threonine‐protein kinase* (*SBK1*). This gene has elevated expression levels in an *O. mykiss* line selectively bred for fast growth and has been linked with growth hormone‐mediated physiological pathways (Cleveland et al., 2020). Although faster growing parr have a higher propensity to adopt residency, in many salmonid species, the relationships between growth/body size and AMTs are not always consistent (reviewed by Dodson et al., 2013; Ferguson et al., 2019). Another gene of interest was *desert hedgehog gene* (*DHH*) that showed higher expression in smolts than in residents. Genes in the hedgehog pathway have been associated with tissue growth/regeneration and developmental changes in teleost fishes (Marí‐Beffa & Murciano, 2010; Sims et al., 2009), including aspects of fin growth (Iovine, 2007). This is noteworthy as migrants go through a series of morphological changes (e.g., body streamlining, changes in fin shape and color) during smoltification.

As physiological influences on, or consequences of, life history decisions, may be mediated by hormones, for example, glucocorticoids (Peiman et al., 2017; but see Jain‐Schlaepfer et al., 2018), or by antioxidant capacity (Birnie‐Gauvin et al., 2017), our results implicate the liver as an important organ involved in the genetic regulation of these processes. This bolster and expands upon earlier work on *S*. *trutta*, which, based on cDNA microarray screens of liver samples, found that almost 21% of 900 screened genes were differentially expressed between sedentary and migratory populations (Giger et al., 2008, but also see Giger et al., 2006). These included the candidate genes *endozepine* and *transaldolase 1* that are involved in lipid metabolism in the liver, which were upregulated in residents, and the constitutive heat‐shock protein HSC70‐1, which was upregulated in smolts (Giger et al., 2008). Our study also found that *transaldolase 1* was upregulated in the livers of residents when compared to the livers of smolts, further supporting these findings.

Aside from differences in genes associated with metabolism, smolt‐biased genes were enriched for Gene Ontology terms associated with cell proliferation and regulation, and response to stimulus. Migrants face a number of threats when moving between environments, including exhaustion, predation, and novel encounters with marine‐based pathogens and disease. While immunity is metabolically costly to constitutively express and actively use, it is hypothesized that migrants will boost their immune potential in preparation for novel pathogen encounter during migration (Buehler et al., 2010; Colgan et al., 2021; Møller & Erritzøe, 1998). Within our study, the most significantly enriched Gene Ontology term for smolt‐biased genes was response to cytokines, which suggests changes in the immune profile of migrants. Cytokines are a broad range of small proteins with immunomodulatory capacities (Kishimoto et al., 1994). While the higher hierarchical GO term for response to cytokines was “response to stimulus,” rather than “immune system process,” cytokines are originally produced in response to antigens, and hence, a link to immune function can be inferred here. Genes falling under the GO term response to cytokines included a number of immune genes with elevated expression in the liver of migrants including *macrophage mannose receptor 1*, *macrophage capping protein*, *LDL receptor‐related protein 2*, *interleukin‐13 receptor*, *lipopolysaccharide‐induced tumor necrosis factor alpha*, and seven genes with putative roles in the complement pathway, a key component of the innate immune system that functions in pathogen removal, opsonization, and promotion of inflammation. While the fish in our study were not maintained in sterile conditions and we would expect some form of background immune expression, differences in immune regulation between alternative life history phenotypes may be associated with preparation for traveling to, and residing in, a marine environment. Alternatively, given the energetic trade‐offs between immunity and reproduction (Roff, 1992), increased immune potential in migrants may be indirectly related to reduced reproductive investment.

In comparison with the liver, we identified fewer differentially expressed genes in the brain indicating unique tissue‐specific profiles that differ between alternative life history phenotypes. This was surprising, given the brain's proposed role as a control center for salmonid AMTs via hormonal cascades (Aubin‐Horth et al., 2009; Dodson et al., 2013). Indeed, previous research on rainbow trout (*Oncorhynchus mykiss*; Hale et al., 2016, 2018; McKinney et al., 2015) identified larger transcriptional differences in the brain between migrants and residents, associated with a range of physiological processes, including phototransduction and circadian rhythm. Here, although we found no enrichment of specific biological processes, there were candidate genes of interest. For example, of the ten genes differentially expressed between migrants and residents, three were annotated as transcription factors, including two zinc finger domain‐containing proteins and transcription Sox18A. Transcription factors, such as *vgll3* and *six6* (Barson et al., 2015; Czorlich et al., 2018), have been linked recently to the age of maturity of *Salmo salar*, demonstrating the importance such genes play in the expression of complex phenotypes. While the exact function of the transcription factors identified in our present study is currently unknown, future functional studies may elucidate their role, if any, in migration within brown trout or other species.

For certain facultative salmonids, such as brown trout and rainbow trout, sexes can differ in their rates of anadromy, with females often gaining more, in fitness terms, from anadromy than males (García‐Vega et al., 2017; Gross et al., 1988; Huusko et al., 2018; Ohms et al., 2013). Within a given life history tactic (e.g., anadromy or residency), the sexes can also differ in behavior, morphology, and physiology; for example, anadromous females may spend longer at sea than anadromous males (Thorstad et al., 2016). Given these expected phenotypic differences between the sexes, we examined genes that were differentially expressed both between males and females and life history phenotypes and found 12 such genes that were differentially expressed in the liver (out of a total of 103 sex‐biased liver genes). While we identified no enrichment of Gene Ontology terms for these 12 genes, one possibility is that they play indirect roles in sex‐specific maturation processes (Rossignol et al., 2011), given that our smolts were all immature and our residents mature. In mice (*Mus musculus*), for instance, glucocorticoid receptor genes in hepatocytes regulate the expression of sets of genes involved in growth and sexual maturation (Engblom et al., 2007). Sex‐biased gene expression in the liver has been previously shown for other salmonids, such as brook charr, *Salvelinus fontinalis* (Sutherland et al., 2019). The lack of observed sex biases in gene expression in the brains of brown trout in our study is surprising given the noted behavioral and hormonal differences between the sexes. Given the cellular complexity of the brain in comparison with the liver, which is more homogenous, our sequencing of the entire brain may have reduced the ability to detect subtle, subregion specific, differences in gene expression between the sexes. Future studies will benefit from more targeted profiling of specific sections of the brain to elucidate fine‐scale differences between the sexes.

Developmental switches in salmonids (Arostegui et al., 2019; Dodson et al., 2013; Mangel & Satterthwaite, 2008; Thorpe et al., 1998) are thought to be triggered by differential expression of one or more “master regulator” genes early in ontogeny (at least in *O. mykiss*); these in turn orchestrate divergent physiological cascades, likely hormonally mediated, that result in AMTs (Aubin‐Horth et al., 2009; Dodson et al., 2013). A major challenge in these studies is in distinguishing cause from effect. On the one hand, observed gene expression differences between tactics could be the actual cause of physiological changes that originally triggered alternative phenotypes, with these “decision genes” potentially remaining switched on (in one tactic) from that point onward. On the other hand, they may simply reflect “downstream” physiological consequences of earlier activation of master regulators that are only expressed during an early sensitivity window (Dodson et al., 2013; Ferguson et al., 2019). Studies that examine gene expression at older ages (e.g., our current study; Aykanat et al., 2011; Giger et al., 2006, 2008; Norman et al., 2014; Seear et al., 2010; Sutherland et al., 2014) might be more likely to identify genes whose differential regulation follows from, rather than causes, the adoption of AMTs. This may be particularly true for organs such as the liver, kidney, or gills, which play diverse roles in physiologically responding to hormonal cascades that likely begin in the brain, for example, via the light–brain–pituitary axis (Ebbesson et al., 2003). Identifying the actual master regulators is much more difficult, and future studies will benefit from time series of gene expression and profiling of associated phenotypic changes across ontogeny (Dodson et al., 2013). Interestingly, in a comparison of two‐year‐old *O. mykiss* smolts against same‐age residents, Baerwald et al. (2016) found 57 differentially methylated regions, over half of which were in transcriptional regulatory regions. This suggests a role for epigenetic regulation of gene expression in mediating AMTs, but it remains unknown when these methylation changes are triggered and by what mechanisms.

## 5 CONCLUSION

Our study represents an important step toward understanding molecular mechanisms underlying alternative life history tactics and the regulatory roles played by different organs. Our list of candidate genes showing differential expression between migratory phenotypes and/or the sexes in brown trout could be considered by future salmonid studies that aim to disentangle molecular processes activating developmental switches from those that follow from their activation. This knowledge should inform efforts to conserve wild or cultured populations of facultatively migratory salmonids and other taxa of ecological or economic importance (e.g., Robinson et al., 2016) and their capacity for evolutionary or plastic responses to anthropogenic change (Mangel & Satterthwaite, 2008; Railsback et al., 2014; Thériault et al., 2008).

## CONFLICT OF INTEREST

The authors declare no conflict of interest.

## AUTHOR CONTRIBUTIONS

**Robert Wynne:** Conceptualization (equal); Data curation (equal); Formal analysis (lead); Methodology (lead); Writing‐original draft (lead); Writing‐review & editing (lead). **Louise C. Archer:** Investigation (supporting); Methodology (supporting); Writing‐review & editing (supporting). **Stephen A. Hutton:** Investigation (supporting); Methodology (supporting); Writing‐review & editing (supporting). **Luke Harman:** Methodology (supporting); Writing‐review & editing (supporting). **Patrick Gargan:** Resources (supporting); Writing‐review & editing (supporting). **Peter A. Moran:** Conceptualization (equal); Investigation (supporting); Writing‐review & editing (supporting). **Eileen Dillane:** Methodology (supporting); Resources (supporting); Writing‐review & editing (supporting). **Jamie Coughlan:** Formal analysis (supporting); Methodology (supporting); Writing‐review & editing (supporting). **Thomas F. Cross:** Writing‐review & editing (supporting). **Philip McGinnity:** Conceptualization (equal); Funding acquisition (supporting); Resources (supporting); Writing‐review & editing (supporting). **Thomas J. Colgan:** Conceptualization (equal); Data curation (equal); Formal analysis (supporting); Methodology (supporting); Supervision (supporting); Writing‐original draft (supporting); Writing‐review & editing (supporting). **Thomas E. Reed:** Conceptualization (equal); Formal analysis (supporting); Funding acquisition (lead); Project administration (lead); Resources (lead); Supervision (lead); Writing‐original draft (supporting); Writing‐review & editing (supporting).

## ETHICAL STATEMENT

All fish were euthanized humanely at the end of the laboratory rearing phase under license. The study and all associated procedures were carried out with ethical approval from Health Products Regulatory Authority (HPRA) Ireland, under HPRA project license AE19130/P034, and HPRA individual licenses AE19130/1087, AE19130/I200, AE19130/I201, and AE19130/I202.

## Supporting information

Table S1

Table S2

Table S3

Table S4

Table S5

## Data Availability

Raw sequence data files are deposited in the NCBI short read archive (BioProject ID: PRJNA670837). Scripts underpinning the analysis of transcript estimation, differential expression, and Gene Ontology term enrichment are available for reuse on GitHub (https://github.com/Joscolgan/salmo_smolt_study). Raw sequence counts for each sample are provided in the [Link], [Link], [Link], [Link], [Link].
